# High-dose intravenous vitamin C may help in cytokine storm in severe SARS-CoV-2 infection

**DOI:** 10.1186/s13054-020-03228-3

**Published:** 2020-08-13

**Authors:** Adriana Françozo de Melo, Mauricio Homem-de-Mello

**Affiliations:** InSiliTox, Pharmacy Department, Health Sciences School, University of Brasilia, Campus Universitario “Darcy Ribeiro”, Brasilia, Brazil

To the editor:

The complete mechanism of action of the SARS-CoV-2 remains unclear. The severity of the disease is probably linked to the “cytokine storm” that leads to the acute respiratory distress syndrome. Cytokine storm is an overwhelming increase of several inflammatory markers at once, and it is a cause of severity in some conditions such as sepsis, acute pancreatitis, and multiple sclerosis, among others.

These conditions, characterized by a variety of inflammatory etiologies, have the common prognostic of hemodynamic abnormalities, multiple organs failure, and high mortality rates. Treatment of cytokine storm associated syndromes usually focuses in this environment: intensive care of hemodynamic parameters, uphold of damaged organs, and supervision of coagulopathy. The main cause of the cytokine induction, however, must yet be treated and is the main reason why in bacterial sepsis antibiotics are used.

High doses of intravenous vitamin C, higher than 6 g per day (however, even 15 g/day, or more, are reported), have demonstrated efficacy in treatment of sepsis and acute pancreatitis [1, 2]. To the best of our knowledge, oral formulations cannot exert the same effects.

In parenteral administration of high doses, vitamin C may decrease several inflammatory parameters (cytokines, cellular response, and homeostatic control), already observed in human and animal models. As adverse effect, ascorbic acid may cause oxalate accumulation in the kidneys that can be prevented with the concomitant use of thiamine.

Recovery Trial [3] recently finished its dexamethasone arm in severe COVID-19 patients and has shown decrease in mortality among patients that requires mechanical ventilation and support of oxygen. We are convinced about a possible synergistic effect of vitamin C and this corticosteroid.

Recently, Dr. Carr commented [4] about a new clinical trial in development in China, using intravenous vitamin C in high dose. This trial, registered by Dr. Peng (Wuhan University), is now closed, since new severe cases became rare in Wuhan. (Confirmed by Dr. Peng by e-mail, even if in clinicaltrials.gov the status of the trial is yet as “recruiting”).

Dr. Hernández and colleagues proposed a protocol including vitamin C in high dose to be used in COVID-19 patients [5]. However, the manuscript is in Spanish, decreasing the accessibility of the information.

The protocol using intravenous high dosage of vitamin C, thiamine and a glucocorticoid (as dexamethasone) seems to be useful, not expensive, with low risk of severe adverse effects. The aim is providing further discussion about the recovery of complicated cases of cytokine storm associated to the SARS-CoV-2.

## Data Availability

N/A
